# Research progress in the risk factors and screening assessment of dysphagia in the elderly

**DOI:** 10.3389/fmed.2022.1021763

**Published:** 2022-11-07

**Authors:** Kerong Chen, Liwei Xing, Bonan Xu, Yi Li, Tianyun Liu, Tingjuan Zhang, Hongping Shi, Hanmei Lu, Wengang Zhou, Jianhong Hou, Hongling Shi, Dongdong Qin

**Affiliations:** ^1^Department of Rehabilitation Medicine, The Third People’s Hospital of Yunnan Province, Kunming, China; ^2^The First School of Clinical Medicine, Yunnan University of Chinese Medicine, Kunming, China; ^3^School of Basic Medical Sciences, Yunnan University of Chinese Medicine, Kunming, China; ^4^Department of Medical, The Third People’s Hospital of Yunnan Province, Kunming, China; ^5^Department of Orthopedics, The Third People’s Hospital of Yunnan Province, Kunming, China

**Keywords:** community-dwelling elderly, dysphagia, screening tool, preventive and therapeutic measures, research progress

## Abstract

With the aging of the population, the incidence of dysphagia has gradually increased and become a major clinical and public health issue. Early screening of dysphagia in high-risk populations is crucial to identify the risk factors of dysphagia and carry out effective interventions and health management in advance. In this study, the current epidemiology, hazards, risk factors, preventive, and therapeutic measures of dysphagia were comprehensively reviewed, and a literature review of screening instruments commonly used globally was conducted, focusing on their intended populations, main indicators, descriptions, and characteristics. According to analysis and research in the current study, previous studies of dysphagia were predominantly conducted in inpatients, and there are few investigations and screenings on the incidence and influencing factors of dysphagia in the community-dwelling elderly and of dysphagia developing in the natural aging process. Moreover, there are no unified, simple, economical, practical, safe, and easy-to-administer screening tools and evaluation standards for dysphagia in the elderly. It is imperative to focus on dysphagia in the community-dwelling elderly, develop unified screening and assessment tools, and establish an early warning model of risks and a dietary structure model for dysphagia in the community-dwelling elderly.

## Introduction

Dysphagia is a process in which food is not delivered safely and efficiently into the stomach due to structural and/or functional impairment of the organs such as the jaw, lips, tongue, soft palate, throat, and esophagus (1). Aging, degradation of physiological function, tumor, stroke, and other nervous system diseases, as well as other underlying diseases make the elderly population susceptible to dysphagia (2). Approximately 8% of the global population suffer from swallowing problems (3), and research shows that the lifetime prevalence rate of dysphagia is 17.10% in the community-dwelling elderly, rising to 52.60% in high-risk populations (4).

At present, research on dysphagia in the elderly has predominantly focused on populations with a high incidence of dysphagia such as inpatients and those that have had a stroke, and the subjects are mostly elderly inpatients. There are limited surveys on the incidence of dysphagia in the community-dwelling elderly, and few studies on the influencing factors of dysphagia in the naturally aging, community-dwelling population. Early screening of populations at high risk of dysphagia is crucial to identify the risk factors of dysphagia, and perform effective interventions and health management in advance, which may reduce the incidence of dysphagia, prevent complications, lower medical burdens, and save medical resources. In the current study, a literature review was conducted on the research progress in the risk factors, screening assessment, preventive, and therapeutic measures of dysphagia in the elderly with the aim providing a reference for the screening and research of dysphagia in the elderly.

## Current epidemiology and hazards of dysphagia

As a syndrome in the elderly, dysphagia has been listed by the World Health Organization (WHO) in the International Classification of Diseases-10 (ICD-10) and the International Classification of Functioning, Health and Disability (ICF) (5), and is a major public health issue worldwide. In the United States, more than half of people older than 60 years have dysphagia, and 60% of residents of nursing homes have experienced dysphagia (5, 6). Nine percent of residents of nursing homes in the Netherlands (7) and 11.4% of people in British communities complained of symptoms of dysphagia (8). In a geriatric Korean community, 33.7% of the population reported symptoms of dysphagia (9). The prevalence of dysphagia is 5.5–12.9% in the elderly in Chinese communities (10), rising to 31.1% in the institutionalized elderly (11). According to one epidemiological survey of 5,943 patients, 2,341 patients (39.4%) had dysphagia, including 51.14% of stroke patients, 34.4% of patients with head and neck cancer, 48.3% of patients with nervous degenerative diseases, and 19.2% of healthy elderly people (12).

Dysphagia hinders the intake of nutrients required by elderly patients, which leads to serious complications such as weight loss, malnutrition, dehydration, aspiration pneumonia, asphyxia, anxiety, and sociopsychological disorders. These complications directly or indirectly influence the long-term prognosis and quality of life (13), prolong hospital stays, and increase hospital readmissions and the risk of death in elderly patients (14). It was reported that over 60,000 people die each year from complications of dysphagia, of which aspiration pneumonia is the most serious complication and the leading cause of death (15). Dysphagia and its complications and sequelae increase the overall utilization of healthcare services and result in a huge consumption of medical resources. It is estimated that annual costs of dysphagia in the US medical care system are between $4 billion and $7 billion US dollars. Moreover, this estimate does not consider indirect costs, such as the economic impact of a patient’s inability to work due to dysphagia symptoms. In addition to medical costs, there are emotional and mental-health-related costs, which seriously impact the quality of life of patients and simultaneously place a heavy burden on the patient’s family, hospitals, and society (16, 17).

## Risk factors for dysphagia in the elderly

Identifying the risk factors of dysphagia in the elderly is the premise and basis for the identification, assessment, and control of these risks. The main risk factors for dysphagia in the elderly are as follows.

### Age

Anatomic and physiological changes in elderly patients are believed to be likely to cause dysphagia (18). The incidence of postoperative dysphagia in patients older than 60 years of age was significantly higher compared with that in patients younger than 60 years, suggesting that age is a risk factor for the development of dysphagia. Another study suggests that although the incidence of diseases that are likely to cause dysphagia, such as stroke, increases with age, the physiological changes, and functional decline that occur in natural aging are associated with the occurrence of dysphagia. With aging, factors such as tooth damage, dull neuroreceptors, decrease in salivary secretion and the elasticity of swallowing organs, and weakening of swallowing muscle strength all increase the risk of dysphagia (3, 19). Thus, even in the absence of underlying diseases, natural aging itself affects swallowing which is supported by the findings of Byeon (4) and Holland et al. (8). Therefore, it is recommended that early screening and assessment of dysphagia is performed to facilitate early intervention in the elderly population older than 60 years.

### Illnesses

The prevalence of dysphagia is 27–64% in stroke patients, and approximately 50% in the acute phase (20). The figure is over 80% in patients with Parkinson’s disease (21) and 38% in those with multiple sclerosis (22). While, it is 34.4% in those with head and neck cancer (23). The incidence of dysphagia in patients with ossification of the anterior longitudinal ligament (OALL) in the neck is influenced by the thickness of osteophytes, the range of cervical motion, and craniocervical alignment, and OALL occasionally leads to dysphagia due to the anterior osteophytes (20). Skeletal muscle loss is also thought to be a possible cause of dysphagia (21).

### Surgical and therapeutic factors

The incidence of dysphagia was reported to be 1–80% following anterior cervical surgery (20, 24). Furthermore, Baron et al. (25–27) found the incidence of transient dysphagia was up to 80% after anterior cervical fusion, and even higher in patients older than 60 years. Dysphagia is one of the most common complications after anterior cervical surgery (28). Severe paralysis and tracheotomy may also be risk factors for dysphagia (29). Oropharyngeal dysphagia (OD) is common in elderly patients with hip fractures and is easily overlooked, predisposing patients to life-threatening postoperative pneumonia. Hip fractures often occur in elderly patients with comorbidities such as stroke or dementia. In addition, the incidence of dysphagia is particularly high in patients with hip fractures due to intraoperative intubation. The prevalence of dysphagia was reported to be 7% in patients with hip fractures but 34% after surgery. These findings suggest that the effects of the disease itself, hospitalization, surgery, and intubation on swallowing function may be temporary, but identification of dysphagia after surgery is necessary to prevent consequences such as pneumonia. These data can help clinicians to manage patients with advanced dysphagia.

### Other factors

Pharmacological factors like opioid and topical steroid (24), psychological factor such as depression (22), as well as mealtime (30), and serum albumin levels (31) are all additional influencing factors of dysphagia.

## Dysphagia screening instruments

There are no unified standards of screening and assessment tools for dysphagia in elderly individuals worldwide, and multiple screening and assessment tools for dysphagia have been developed according to individual situations. Screening and assessment tools are reviewed in the current study. Table 1 details the name of the assessment tool, the country and intended populations, main indicators, methods and characteristics.

**TABLE 1:** Common screening and assessment tools for dysphagia.

Test	Country of origin	Indicators	Methods/Descriptions	Characteristics	References
Videofluoroscopic swallowing study (VFSS)	USA	Swallowing process	Patients were given the liquid and food, seat in chairs for a video lateral view of oral, pharyngeal, and upper esophageal phases of swallowing	Gold standard for screening; not suitable for preliminary screenings due to facility requirements and radiation	van den Engel-Hoek et al. (34)
Water swallowing test (WST)	Japan	Swallow function	Patients swallowed warm water and were monitored for the presence of a choking cough	Convenient and rapid, wide applications, but the specificity is low	Osawa et al. (32)
Repeated salivary swallowing test (RSST)	Japan	Elevation range of Adam’s apple and hyoid bone	Counting the frequency of swallows over 30 s by inspection and palpation of the prominentia laryngea	Rapid and simple; strong subjectivity	Hongama et al. (47)
Simple coughing test (SCT)	Japan	Cough	Patients orally inhaled a mist of 1% w/v citric acid-physiological saline and were asked to inhale deeply through the mouth according to verbal instruction until the first cough occurred	Easy to administer	Sato et al. (48)
Volume-viscosity swallowing test (V-VST)	Spain	Cough, vocalization, blood oxygen saturation, swallowing times	Solutions were prepared 10 min prior to measurement. Boluses of 5, 10, and 20 mL of each viscosity series were offered to patients with a syringe during V-VST	Safe, effective, convenient, and rapid	Clavé et al. (49)
Munich dysphagia test – Parkinson disease (MDT-PD)	Germany	Food and fluid intake, concomitant symptoms, etc.	The final 26-item MDT-PD consists of four sub-scales, and the time to fill out the questionnaire is about 10 min	Highly sensitive and specific; time-consuming	Simons et al. (50)
Toronto bedside swallowing test (TOR-BSST)	Canada	Swallowing ability	The TOR-BSST is a one-page, double-sided form. Page one consists of the screening tool and page two contains standardized instructions for the administration of the tool	Rapid and convenient	Martino et al. (51)
Yale swallowing protocol	USA	Cough, vocalization	Uninterrupted drinking (assistant or independent) of 3 ounces of water from a cup and without coughing	Highly sensitive and scientific	Suiter et al. (52)
Eosinophilic esophagitis activity index (EEsAI)	USA	Duration and whole process of food ingestion	Patient-reported outcome (PRO) were assessed separately from items measuring biologic activity	Comprehensive; time-consuming	Schoepfer et al. (53)
The gugging swallowing screen (GUSS)	Austria	The food swallowing process	Part 1: Preliminary assessment: Indirect swallowing test. and part 2: Direct swallowing test	Simple, safe, repeatable, but unable to reflect specific conditions	Trapl et al. (54)
Functional oral intake scale (FOIS)	USA	Swallowing ability	Firstly, initial scale development and item selection, face validity, interrater reliability, and consensual validity; and secondly, criterion validity, cross-validation, and evaluation of sensitivity to expected change in functional performance	Simple and clear; limited scope of use	Crary et al. (55)
Dysphagia risk assessment for community-dwelling elderly (DRACE)	Japan	−	A questionnaire including 18 items based upon physical symptoms of chewing and swallowing disorders during the previous year and 3-point scale was applied according to severity of the events	Easy to administer; low accuracy and reliability	Miura et al. (56)
Modified mann assessment of swallowing ability (MMASA)	USA	Consciousness, respiration, and tongue muscle movement, etc.	Items were: alertness, cooperation, respiration, expressive dysphasia, auditory comprehension, dysarthria, saliva, tongue movement, tongue strength, gag, voluntary cough, and palate movements	Easy to administer, rapid	Antonios et al. (57)
Swallowing outcome after total laryngectomy (SOAL)	UK	Subjective feeling of swallowing, dysphagia with various forms of food, food retention	Internal consistency, group discrimination and relation with an instrumental measure of dysphagia were addressed to assess three important aspects of validity	Easy to administer	Govender et al. (58)
Metro dysphagia screening (MDS)	USA	Vocalization, drooling, cough	A screen that was easy for nursing to apply and readily detect dysphagia	Easy to administer, rapid	Schrock et al. (59)
Preclinical dysphagia screening tool (PRO)	Switzerland	Swallowing function, physical function, and cognitive factors	The survey contained 34 items representing five broad categories drawn from the conceptual framework	Comprehensive; limited scope of use	Madhavan et al. (60)
Post-extubation dysphagia screening instrument (PEDS)	USA	Consciousness, respiration, symptoms, and tubing sets	The screening tool consists of 5 sections of nursing assessments: SLP evaluation, level of alertness, symptoms and tubes, new-onset aspiration symptoms, and contraindications for trial feedings	Rapid, simple and reliable screening tool; indicated only for inpatients after extubation	Johnson et al. (61)
Acute stroke dysphagia screening	USA	Consciousness, vocalization, swallowing ability	The first section ensured that the patient was physically able to participate in the screening and included level of alertness/responsiveness and the ability of the patient to be positioned upright with some degree of head control. The second section evaluated voluntary cough, salivary management, the ability to lick the top and bottom lip, respiratory function, and vocal quality	Of high reliability, validity, and sensitivity	Edmiaston et al. (62)
Dysphagia symptoms questionnaire (DSQ)	USA	Swallowing after wakening in the morning	(1) “Since you woke up this morning, has food gone down slowly or been stuck in your throat or chest?” and (2) “For the most difficult time you had swallowing food today, did you have to do anything to make the food go down or take action to get relief?”	Easy to administer, rapid; too simple and low accuracy	Dellon et al. (63)
Swallowing disturbance questionnaire (SDQ)	Israel	Oropharyngeal swallowing disorder and frequency of occurrence	Swallowing disturbance questionnaire including 15 questions and was rated by a four-point (0 –3) scale	Concise, easy to administer; limited range of use	Manor et al. (64)
The DYMUS (questionnaire for the assessment of dysphagia in multiple sclerosis)	Italy	Ability to swallow solids and liquids	The questionnaire was initially composed of 15 items. All the answers were dichotomous, coded as 1 or 0, depending on the presence or the absence of the event	Simple, convenient, and rapid to administer	Bergamaschi et al. (65)
Mayo dysphagia questionnaire (MDQ)	Germany	Swallowing, heartburn, esophageal reflux	The MDQ, a 28-item instrument, took an average of 10 min (5–30 min) to complete	Simple, effective; time-consuming	McElhiney et al. (66)
Esophageal symptoms questionnaire (ESQ)	USA	Dysphagia, sensation of obstruction, reflux symptoms	ESQ included 19 items measured in both frequency and severity of symptoms resulting in 38 total items	Accuracy depends on subjective judgment	Kwiatek et al. (67)
Brief esophagus dysphagia questionnaire (BEDQ)	USA	Frequency, severity, and impact of dysphagia	A 10-item self-report measure of dysphagia symptom frequency (5 items), severity (3 items), and impaction (2 items)	Time-consuming, a limited scope of use	Taft et al. (68)
Dysphagia handicap index (DHI)	USA	Physical, functional, and emotional effects of dysphagia	Statements were 60 in number and used to ensure that the scale had both content and face validity. The 60 statements were sorted into three subscales based on their contents	Easy to administer, rapid	Silbergleit et al. (69)
Four-question test (4-QT)	Hong Kong, China	Eating and changes in eating	(1) Do you cough and choke when you eat and drink? (2) Does it take longer to eat your meals than it used to? (3) Have you changed the type of food that you eat? (4) Does your voice change after eating/drinking?	High sensitivity; low specificity	Tsang et al. (70)
Eating assessment tool (Eat-10)	USA	Clinical features, psychological feelings, influences on social intercourse, etc.	Each clinician was asked to review the dysphagia literature from his or her own clinical experience, examine other dysphagia questionnaires, and contribute 10 questions he or she deemed to have excellent face validity to the original survey. All items were arranged in a 5-point likert scale	Easy to administer, wide application, rapid; not indicated for fasting patients	Belafsky et al. (71)

In general, there are pros and cons to the various screening tools, and a widely accepted, perfect assessment tool is currently lacking. Munich Dysphagia Test–Parkinson Disease (MDT-PD) includes difficulty in swallowing general food, dysphagia unrelated to food, burden related to dysphagia, and health problems caused by dysphagia. There are 4 major parts, 26 items, and a score of < 3.65 indicates no symptoms of dysphagia. A score of 3.65–4.78 indicates early oropharyngeal dysphagia, and a score of ≥ 4.79 indicates that the patient is at risk of aspiration. In addition, 3-ounce water swallow test, repeated salivary swallowing test, simple swallowing provocation test (S-SPT) and other tests are also applied. The water swallowing test WST (32) is the most classical and most commonly used assessment methods in clinical practice, but the specificity is low, and latent aspirations cannot be effectively predicted for it could only identify the inability of safety concerns when swallowing water in patients with stroke. In addition, the risk of aspiration pneumonia from the use of these methods may adversely affect patient prognosis (33). Videofluoroscopic swallowing study (VFSS) allows real-time visual observation of the swallowing process and qualitative and quantitative analysis, which is regarded as an ideal method and gold standard for diagnosis of dysphagia (34). However, VFSS has drawbacks such as being time-consuming and complicated and causing exposure to radiation, consequently VFSS is unsuitable for early screenings.

In addition, currently available screening instruments for dysphagia are mainly aimed at inpatients with certain diseases or other unique conditions, especially elderly patients, and these tools cannot meet the screening needs of elderly people dwelling in nursing homes and communities.

## Preventive and therapeutic measures

Due to increased risk of dysphagia in the elderly which is probably the result of complicated risk factors as forementioned, there is clearly a need for preventive and therapeutic measures for dysphagia in the elderly which are individualized according to their specific risk factors.

### Preventive measures

Prophylactic swallowing exercises can avoid periods of nothing per oral (NPO) which is the commonest preventive measure based on the rule of “use it or lose it” (35). Study has shown that prophylactic exercises may result in maintenance of oral and oropharyngeal musculature, improved swallowing function, and less dysphagia-related aspiration pneumonia (36). Interventions to prevent dysphagia in older adults living in nursing homes included more bedside evaluation, modification of dietary, creating an appropriate environment for swallowing, providing appropriate feeding assistance, appropriate posture or maneuver for swallowing, appropriate rehabilitation program, medication treatment, and stimulation treatment. Among them, modification of dietary was the most frequently used intervention to prevent or reduce aspiration (37). Saliva aspiration prevention like oral anticholinergics, transdermal anticholinergics, intravenous anticholinergics, and salivary gland irradiation, as well as active dysphagia revalidation including bedside swallow exercises, swallow training with electrical stimulation and swallow training with surface electromyographic biofeedback were proven to be effective preventive measures for dysphagia (38).

### Therapeutic measures

The primary goal of therapy is an adequate diet without any risk of aspiration, such as utilizing fluid adaptation with thickeners to avoid impaired safety, and postures and maneuvers to compensate biomechanical alterations are also generic protocols (39). Fluid and nutritional adaptation was proven to be therapeutic in older patients with dysphagia by reducing the prevalence of laryngeal vestibule penetrations and tracheobronchial aspirations (40). Due to its unpleasant taste, however, which many patients find problematic to swallow on a daily basis, thus results in low compliance (41). The minimally massive intervention (MMI) was developed to reduce nutritional and respiratory complications in older hospitalized patients with dysphagia (42). The MMI consists of the following steps: (1) dysphagia evaluation with a clinical tool and adaptation of fluids to avoid impaired safety of swallow, (2) nutritional evaluation and a triple adaptation of food with high-calorie, high-protein and high-vitamin to improve patient nutritional status, and (3) oral health and hygiene evaluation and treatment to avoid respiratory pathogen colonization of the oral cavity (43). Preliminary results suggest that the MMI might become a simple and cost-effective strategy to reduce dysphagia complications in the geriatric population with an acute disease admitted to a general hospital (44).

New treatments based on stimulation of sensorial and motor neural pathways promote swallowing function recovery rather than compensating it. Intrapharyngeal or transcutaneous neuromuscular electrical stimulation, as well as chemical or pharmacological stimulation using TRPV1 (transient receptor potential vanilloid 1) agonists like capsaicin and piperine, which heighten sensory stimuli to the afferent pathway of deglutition, are the peripheral stimulation techniques that have got the most attention (45, 46). Because there are few studies and small patient samples, there is minimal scientific evidence for these therapy approaches, but initial findings are intriguing and promising. As a result, therapies for dysphagia in elderly patients are quickly transitioning from compensatory to therapeutic approaches that encourage the restoration of swallow function.

## Summary

Considering the hazards of dysphagia, screening of dysphagia is crucial for elderly patients. Risk factors including age, illnesses, surgical, and therapeutic factors are the premise and basis for the diagnosis, assessment, and control of dysphagia in the elderly. Screening and assessment tools reported in the last two decades indicated that a widely accepted, perfect assessment tool is yet currently lacking. Some compensatory measures and new treatments based on stimulation of sensorial and motor neural pathways can promote swallowing function recovery. More efforts should be focused on early identification and effective prevention and rehabilitation. Reduced morbidity in elderly populations may be achieved by addressing issues like the most efficient and effective ways to detect malnutrition and dysphagia in high-risk patients and community-dwelling elderly persons.

## Perspectives

With the increasing incidence of dysphagia, there is an urgent need to explore barriers and facilitators of different risk factors, screening tools and therapeutic strategies in detail. Limitations still exist throughout the available research including short duration of many interventions, variations in types of participants, differences in the methods used to diagnose dysphagia, poor design, and poor interpretation of results. Many trials that were identified had small sample sizes and lacked the ability to be generalized to a wider population. Dropout rates and lack of true randomization of trials also weaken the available research. To further clarify different risk factors, screening tools and therapeutic strategies underlying dysphagia in the elderly, a need remains for future large-scale multi-center randomized controlled trials, risk prediction model of dysphagia in elderly patients and in-depth mechanism studies, with the aim of minimizing the occurrence of dysphagia in elderly populations.

## Author contributions

All authors listed have made a substantial, direct, and intellectual contribution to the work, and approved it for publication.
